# Nitric Oxide: Exploring the Contextual Link with Alzheimer's Disease

**DOI:** 10.1155/2016/7205747

**Published:** 2016-12-21

**Authors:** Nicholas Asiimwe, Seung Geun Yeo, Min-Sik Kim, Junyang Jung, Na Young Jeong

**Affiliations:** ^1^Department of Biomedical Science, Graduate School, Kyung Hee University, 26 Kyungheedae-ro, Dongdaemun-gu, Seoul 02447, Republic of Korea; ^2^Department of Otorhinolaryngology, H & N Surgery, College of Medicine, Kyung Hee University, 26 Kyungheedae-ro, Dongdaemun-gu, Seoul 02447, Republic of Korea; ^3^East-West Medical Research Institute, Kyung Hee University, 26 Kyungheedae-ro, Dongdaemun-gu, Seoul 02447, Republic of Korea; ^4^Department of Applied Chemistry, College of Applied Sciences, Kyung Hee University, Deogyeong-daero, Giheung-gu, Yongin-si, Gyeonggi-do 17104, Republic of Korea; ^5^Department of Anatomy and Neurobiology, College of Medicine, Kyung Hee University, 26 Kyungheedae-ro, Dongdaemun-gu, Seoul 02447, Republic of Korea; ^6^Department of Anatomy and Cell Biology, College of Medicine, Dong-A University, 32 Daesingongwon-ro, Seo-gu, Busan 49201, Republic of Korea

## Abstract

Neuronal inflammation is a systematically organized physiological step often triggered to counteract an invading pathogen or to rid the body of damaged and/or dead cellular debris. At the crux of this inflammatory response is the deployment of nonneuronal cells: microglia, astrocytes, and blood-derived macrophages. Glial cells secrete a host of bioactive molecules, which include proinflammatory factors and nitric oxide (NO). From immunomodulation to neuromodulation, NO is a renowned modulator of vast physiological systems. It essentially mediates these physiological effects by interacting with cyclic GMP (cGMP) leading to the regulation of intracellular calcium ions. NO regulates the release of proinflammatory molecules, interacts with ROS leading to the formation of reactive nitrogen species (RNS), and targets vital organelles such as mitochondria, ultimately causing cellular death, a hallmark of many neurodegenerative diseases. AD is an enervating neurodegenerative disorder with an obscure etiology. Because of accumulating experimental data continually highlighting the role of NO in neuroinflammation and AD progression, we explore the most recent data to highlight in detail newly investigated molecular mechanisms in which NO becomes relevant in neuronal inflammation and oxidative stress-associated neurodegeneration in the CNS as well as lay down up-to-date knowledge regarding therapeutic approaches targeting NO.

## 1. Introduction

Nitric oxide (NO) is an endogenously synthesized free radical and a member of the gaseous signaling molecules widely known as gasotransmitters. It participates in a host of autocrine and paracrine bodily physiologies ranging from cardiovascular homeostasis to modulating immunological and neurological functions. NO tends to differ from its typical neurotransmitter counterparts in a number of ways. For example, unlike the conventional neurotransmitter signaling pathways that entail cognate receptor binding, NO directly modifies its intracellular targets due to the fact that it passively can permeate the cellular membrane [1]. In the cardiovascular system, this signaling molecule is involved in the relaxation of smooth muscles of the vascular tissue [2] and partakes in neurotransmitter release from motor nerve endings. Apparently, it also can mediate synergistic, differing, and sometimes opposing biological effects, which may be due to a number of factors at play including the second messenger through which it is mediating its physiological effects [3, 4]. With respect to vasodilation where its physiological role has been thoroughly elucidated, NO signals by interacting with soluble guanylyl cyclase (sGC) which results in upregulation of intracellular cyclic guanosine monophosphate (cGMP) levels [5–8].

For many years, NO had been known pretty much for its noxious effects to the body [9]. As a result, its potential beneficial roles were of petite significance to the scientific sphere. However, the past few decades have witnessed an explosion in published data about its multiple physiological roles in the normal functioning of the body. First recognized for its relaxing properties by Furchgott and his colleague in 1980 as an endothelium-derived relaxing factor (EDRF) [10], it did not take long before NO, the first of the gasotransmitters to be studied in detail, was implicated in CNS physiology [11]. Consequently, its unregulated biosynthesis would eventually be appreciated in neurodegenerative disorders. Attempts to understand the mechanisms through which NO induced neurotoxicity accentuated the importance of ROS and RNS. Interestingly, evidence pins NO and oxidative stress to both early and late stages of neurodegenerative disorders, as well as promoting their progression [12, 13].

During oxidative/nitrosative stress, NO and its reactive secondary metabolites oxidize/nitrosate various molecular targets such as proteins, lipids, and nucleic acids, potentially causing ruinous cellular disorders [14, 15].

AD is an enervating neurodegenerative disorder whose underlying principal etiology is yet to be made definite. Mounting evidence suggests the oxidative stress and inflammation as important pathophysiological mechanisms in the pathogenesis of AD. Moreover, NO seems to be the heartbeat of oxidative stress-associated effects manifested in AD. Therefore, in this review, we essentially discuss the roles played by oxidative stress-associated neuronal inflammation in neurodegenerative disorders, with a particular focus on AD. We explore recent experimental data that relate to molecular pathways modulated or altered by NO in the context of this form of dementia. We also lay down pertinent focal knowledge points regarding therapeutic approaches targeting NO and both its upstream and its downstream pathways.

## 2. Biosynthesis of NO

NO is a small unstable and highly lipophilic gas endogenously synthesized by several cell types and exerts multiple biological regulatory roles at a local level in inflammation, nervous and cardiovascular systems, and bone resorption [16–18]. NO and L-citrulline are the end products of a reaction catalyzed by a family of homodimeric and heme containing nitric oxide synthases: the inducible NO synthase (iNOS) and the constitutively expressed neuronal NO synthase (nNOS) and the endothelial NO synthase (eNOS). Nicotinamide adenine dinucleotide phosphate (NADPH), tetrahydrobiopterin (BH4), and oxygen (O_2_) serve as cofactors in this pathway.

nNOS is expressed in neuronal cells and regulates the release of neurotransmitters [19]. Together, eNOS and nNOS, whose activation is dependent on Ca^2+^/calmodulin, are produced in the brain under physiological conditions. On the contrary, iNOS is principally produced by astrocytes, microglia, and blood-derived macrophages in response to foreign bodies and tissue damage and so is a chief physiological step towards successful arrest of the invading offender by the innate immune system. In addition, reactive microglia and astroglial cells tend to generate iNOS resulting in the biosynthesis of relatively high quantities of NO [10].

NOSs expression is highly regulated by a number of such mechanical factors as fluid shear stress and intrinsic signals such as increased intracellular Ca^2+^ and interaction with substrates and cofactors, as well as adaptor and regulatory proteins, protein phosphorylation, cytokines, and endotoxins [20, 21]. Therefore, any disparities in the abovementioned regulatory factors could prompt down- or upregulation of NOSs, consequently leading to various pathological ramifications such as neurodegeneration.

## 3. Role of NO in Inflammation and Oxidative Stress

### 3.1. iNOS and Inflammation

**Figure 1:**
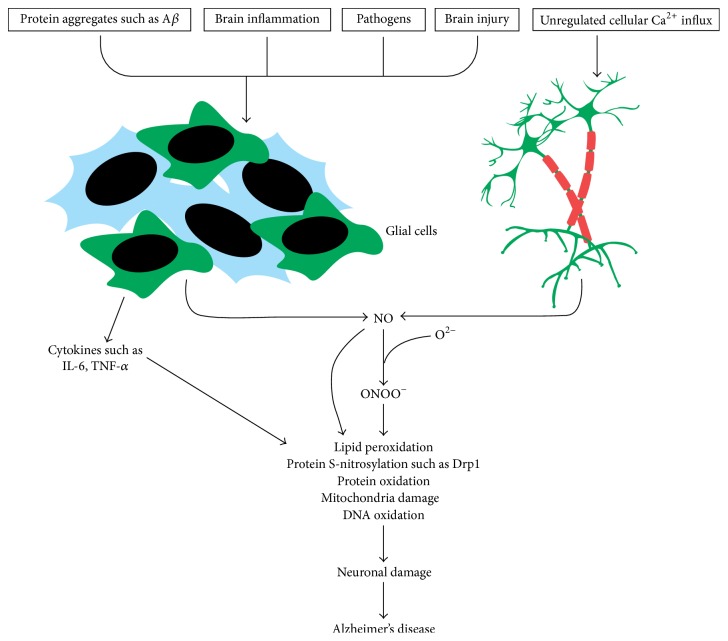
Hypothetical illustration of nitric oxide (NO) and how it is linked to Alzheimer's disease. (A) Protein aggregates, invading pathogens, and cellular death due to inflammation and injury are sensed by resident immune cells such as the glia and astrocytes and become activated. These cells then secrete induced nitric oxide synthase (iNOS), leading to the generation of NO. NO reacts with superoxide to form peroxynitrite. Peroxynitrite oxidizes various macromolecules such as DNA, lipids, and proteins. NO also directly can nitrosylate macromolecules without requiring an intermediary molecule. It also inactivates respiratory enzymes leading to a reduction in ATP production, hence disrupting bioenergetics. (B) Glutamate acts on N-methyl-D-aspartate receptors and triggers inflow of calcium ions. In Alzheimer's disease, ryanodine receptor expression is increased resulting in upregulated calcium ions influx. This activates nNOS neurons to express nNOS, leading to the synthesis of NO. Sustained calcium inflow results in increased NO synthesis leading to oxidative stress/nitrosative stress.

NO plays important roles in pathologies like hypertension, stroke, and neurodegenerative diseases [22]. Glial cells, which are the crucial resident immune cells of the CNS, serve to detect and clear not only cellular fragments following an injury, but also invading pathogens and protein aggregates such as A*β* [23–27]. These nonneuronal cells, immune cells alike, deploy pathogen recognition receptors (PRRs) and damage associated receptors (DAMPs) to sense these microbes and molecules which are foreign and unnatural to the normal functioning of the body. Evidence pinning the reactivation of glial cells by A*β* deposition has been confirmed immunohistochemically. Both reactive and inactive microglia and astrocytes have been reported to be in apposition with the plaques indicating their potential interaction with A*β* plaques [28, 29] (Figure 1). Upon arresting the offender, a number of such anti-inflammatory mechanisms as secretion of IL-10 and TGF-*β* and production of AMP by encephalitogenic and meningeal Treg cells and Th2 cells are initiated to downregulate microglial inflammatory reactions bringing the inflammatory insult to a resting state [30, 31]. However, chances are that the proinflammatory molecules synthesis may persist.

Among the biomolecules produced by microglial cells during an injury or when responding to foreign antigens is NO, a very potent molecule which is neuroprotective at physiological levels and neurotoxic when produced in profuse quantities [18]. In a chronic inflammatory response due to a persistent infection or even due to continual deposition of inflammation triggering mediators, which in this context may be A*β* (both the soluble and the fibrillar forms) in AD, there is a sustained high level of induced NO (iNOS) resulting in increased levels of NO [32–35].

### 3.2. NO and Oxidative Stress-Associated Lipid Peroxidation

NO interacts with multiple effector molecules; for example, NO reacts with free radical superoxide (O_2_^−^) forming peroxynitrite (ONOO^−^) and peroxynitrous acid lowering its bioavailability. Upregulated levels of ONOO^−^ have been reported in AD animal models, particularly one designed to overexpress the amyloid precursor protein (APP) [36]. This highly reactive nitrogen species can cause deleterious oxidative damage to membrane unsaturated fatty acids such as polyunsaturated fatty acids (PUFAs) [37, 38].

Fatty acid peroxidation releases chemically reactive aldehydes: malondialdehyde (MDA), acrolein, 4-hydroxy-2-hexenal (HHE), and 4-hydroxy-2-nonenal (HNE) [39, 40]. HNE, a hydrophobic molecule that has garnered attention, is essentially located in membranes and reacts with membrane proteins forming adducts on neuronal cells [41]. This sets HNE on course to potentially disrupt the movement of signals along the neurons as well as induce neuronal injury and in the end cause cell death. HNE also reacts and adds covalent modifications to A*β*, leading to the formation of cross linkages and hence aggregation, a distinctive characteristic of Alzheimer's disease [42]. Acrolein, the other metabolite of lipid peroxidation, has its own share of noxious ramifications such as DNA oxidative stress and protein adduction, mitochondrial biogenetics disruption, and membrane disruption [43]. Therefore, upregulated levels of NO as a result of inflammation not only could exacerbate the progression of the disease but also could serve to actuate the genesis of other neuronal and nonneuronal pathologies such as cancer.

Proteins equally are possible targets of macromolecular modification by NO and its tributary metabolites. This alteration process is well known as S-nitrosylation and it pertains to the addition of nitrosative species to the tyrosine and cysteine residues. Piling evidence suggests that overly generated NO can induce S-nitrosylation directly or through its secondary metabolite peroxynitrite of certain target proteins. In Alzheimer's disease, noxious NO levels were found to nitrosylate dynamin-related protein 1 (Drp1), which is a crucial component protein involved in the mitochondrial fission-fusion process. This leads to mitochondrial fragmentation and synaptic impairment [44–46].

### 3.3. NO and Oxidative Stress-Associated S-Nitrosylation

Numerous data point out the deposition of misfolded proteins in AD patients [47], which could be attributed to failed systems in posttranslational modifications (PTMs). Moreover, it is becoming clear that PTMs such as S-nitrosylation rather contribute to the formation of misfolded proteins leading to their accumulation in tissues. A case in point is an endoplasmic reticulum (ER) chaperon, protein disulfide isomerase (PDI), which participates in protein folding and ER stress [48]. Uehara et al. demonstrated that direct transfer of NO group to cysteine thiols of PDI in HEK-293 cells, which impeded its catalytic activity, led to the accretion of polyubiquitinated proteins and activated the unfolded protein response [48]. This inhibition, however, was inhibited by a specific NOS inhibitor. It was also confirmed later that S-nitrosylated PDI could colocalize with tau in neurofibrillary tangles (NFTs) [49, 50]. Accumulation of such proteins can lead to apoptotic neurotoxicity and hence neuronal cell loss.

Moreover, ONOO^−^ is suggested to cause protein aggregation still through nitrosylation of membrane protein thiols in mitochondria. This leads to the formation of pores which would compromise the structural and organizational integrity of the mitochondrial membrane, hence resulting in efflux of mitochondrial contents such as apoptosis inducing factors, consequently leading to apoptotic neuronal loss [51]. In addition, ATP production is severely compromised leading to inevitable cell death as reviewed elsewhere [51].

### 3.4. eNOS and Inflammation

eNOS, the isoform generated by endothelial cells, is constitutively generated and regulates vascular tone and growth [52]. Both the deficit and the overexpression of this enzyme have been implicated in several neuropathies. NO, by suppressing cell adhesion promoting vascular cell adhesion molecule-1 (VCAM-1) and intracellular adhesion molecule-1 (ICAM-1) [53], inhibits platelet and leukocyte adhesion to the endothelium, ultimately inhibiting proinflammatory cells and other proteins from continuously homing to the inflamed tissue [54, 55]. As such, this would help to resolve proinflammatory reactions. However, in aging people, the vascular output in the CNS gradually declines, and the endothelial functioning in the context of NO output is not an exception either, with the ultimate outcome of reduced NO bioavailability [54, 56]. Depleted secretion of NO has been reported to promote vascular wall structural changes in mice overexpressing APP [54, 57]. These structural alterations were found to be associated with decreased levels of endothelial NO synthase, increased serum vascular endothelial growth factor, and collagen-I and collagen-IV levels. This triggered membrane thickening in the cerebrovascular system in AD patients, as reviewed elsewhere [54], and sustained a chronic inflammation in the brain tissue. In summary, vascular output deficits as a result of advanced age, collagen deposition, and vascular wall thickening could compromise the supply of nutrients and molecular oxygen to the vital components of the brain facilitating brain damage and aggravate AD by lowering the ability to clear A*β* plaques.

Accumulating evidence suggests also that NO competes with oxygen at cytochrome oxidase and this disrupts mitochondrial energy dynamics [58]. Impaired energy production and disrupted mitochondrial function leading to a disparity in the ability to counteract the ROS will inevitably lead to neuronal cellular death. Therefore, NO participates in a whole host of signaling pathways under both normal physiological and pathological conditions. Both underexpression (low bioavailability) and overproduction of this critical molecule compromise the homeostatic balance of its molecular targets and itself. Hence, all put together, the ultimate upshot heavily inclines on its concentration at a given locale, the underlying mechanisms or conditions triggering its release, and the different kinds of downstream molecular targets it interacts with in a given signaling pathway [59], as well as the site of action.

## 4. Other Isoforms of NOS: Roles in Neurodegeneration

Studies continue to depict, of the three isoforms, iNOS to be the primary contributor to the development of neurodegeneration and/or AD. However, the literature remains not only inconclusive but also debatable [60]. A previous study by Kummer et al. showed that nitrosylation of A*β* at tyrosine-10 is regulated by NOS2 (iNOS) in APP/PS1 mice and is an early stage in the development of senile plaques and that plaque formation was ameliorated by NOS2 gene knockout [61]. By contrast, Colton et al. demonstrated that NO synthase 2 (NOS2) ablation in an Alzheimer's mouse model APP Swedish mutation (APPsw) amplified insoluble *β*-amyloid peptide levels, neuronal degeneration, caspase-3 activation, and tau cleavage [62]. Such conflicting data could be due to a number of factors, especially in the design of experiments such as mouse models being used, conditions in which experiments are set up, and a combination of genes that are manipulated so as to assess their phenotypes. These findings indicate that iNOS and subsequently NO synthesis could have multiple pathways and critical points in which NO becomes pathologically relevant in the initiation and advancement of neurodegeneration. This creates a knowledge gap with respect to what conditions and experimental settings allow for manipulation of iNOS for therapeutic strategy formulation.

But it is not just iNOS that is the center of attention. The other isoforms (eNOS, nNOS) alike, though to a less clear extent, are being investigated for their presumptive involvement in the pathology of neurodegeneration. Given the fact that all forms are heavily involved in multiple brain physiological events such as vasodilation, neuroinflammation, and neuromodulation [63], digression from the norm clearly is bound to alter the functioning of the brain's signaling mechanisms.

Physiologically, nNOS is activated by calcium entry into the cell resulting in NO synthesis. NO goes ahead to influence neurotransmitter release. In AD, calcium release from both intracellular calcium stores and extracellular sources has been shown to be dysregulated starting from as early as the onset of the disease [64, 65]. Several mechanisms are involved in this calcium imbalance. Chakroborty et al., by using a 3xTg-AD mouse model, demonstrated an increase in ryanodine receptor (RyR) expression which caused a spike in calcium-induced calcium release (CICR) in the early stages of AD. But this increase played a compensatory role in synaptic deficiency [64]. On the contrary, in late AD, increased nNOS activation as a result of RyR- induced calcium release gave rise to oxidative- and nitrosative stress-associated macromolecule damage, with mitochondrial energetics disruption eventually leading to synaptic loss and neuronal cellular death [64].

eNOS just as its family members has been implicated in neurodegenerative disorders. Experiments in late middle aged (LMA) eNOS deficient mice (LMA eNOS−/−) revealed a barrage of pathological changes [60]. Austin and her colleagues went on to observe an increase in APP and *β*-secretase (BACE1) expression and increased accumulation of A*β* in the hippocampus compared to age-matched controls. The team also noted increased microglial activation and heightened secretion of inflammatory agents [60]. Thus, from the roles of eNOS such as vasodilation, we put forward a possible explanation for such sequelae in the eNOS deficient mice. eNOS deficiency could result in typical hypoperfusion. As a result, blood flow is reduced dramatically and so clearance of amyloid beta protein could be affected greatly. With accumulating A*β* protein comes along the activation of microglia and initiation of inflammatory responses.

NO-soluble guanylyl cyclase-cyclic guanosine monophosphate (cGMP) transduction pathway has also been known to modulate axonal growth and nerve regeneration [66]. As far as degeneration due to nerve injury is concerned, nNOS and eNOS have also been found to have a role to play. Mice with both genes ablated were found to be resistant to exogenously induced demyelination [67]. In iNOS gene knockout mice, there was delayed degradation and regeneration of myelin sheath after chronic constriction injury (CCI) and complete nerve crash and transection [68, 69]. However, the investigators found that demyelination (the regeneration process) in nNOS, iNOS, and eNOS lacking mice was relatively slow [67]. These data clearly highlight the crucial role of NO in the remyelination process. As NO deficiency has emphasized its importance in nerve regeneration, upregulated levels have been shown to have direct neuronal damage, with particularly the axons being the target of NO-cGMP transduction pathway [70]. Therefore, these findings seem to suggest that neurodegeneration is independent of the isoform at play. In any imbalance, be it elevation or underregulation of any isoform, the eventual sequel in part is due to the level of bioavailability of NO and not wholly the type of the enzyme. However, the source of NO cannot be entirely underappreciated as it will definitely influence the tissue concentrations.

## 5. NO and Alzheimer's Disease (AD)

Alzheimer's disease (AD) is a form of dementia diagnosed among the elderly. It typically is characterized by extracellular deposits of proteolytically APP-derived *β*-amyloid (A*β*) and deposition of intracellular neurofibrillary tangles (NFTs) composed of highly phosphorylated tau protein [71], leading to the commencement of synaptic and neuronal loss [72].

Furthermore, the cytosolic APP domain (AICD) generated by *α*-, *β*-, and *γ*-secretase activity is involved in the pathology of the disease. This protein interacts with a number of genes that regulate actin organization [73] and also has been reported to upset mitochondrial energy dynamics [74]. AICD, which also interacts with FE-65 protein through a phosphotyrosine binding domain (PTB), translocates to the nucleus to form aggregated protein complexes called nuclear spheres. These structures were recently found to be upregulated in the brain tissue, particularly the AD-specific degenerating cell type, of AD patients compared to age-matched controls [75], yet adding another piece to the puzzle. Phosphorylation of AICD at T668 has been shown to aid its nuclear migration, consequently effecting the activation of GSK-3*β* activation and scaling up tau phosphorylation [76].

The potential participation of these protein aggregates in AD pathogenesis has been and still is the widely pursued line of research into the pathology of AD; until recently, a growing body of evidence increasingly implicated the role played by inflammation [77]. The primary cells involved in neuronal inflammation are the neurons, endothelial cells, the nonneuronal astrocytes, blood-derived macrophages, and the microglia, and each cell type's dynamics and biological actions are altered in Alzheimer's brain [63]. During an inflammation, NO synthesis by iNOS is upscaled and it is this particular event that is considered to be a major contributor to oxidative stress-associated neurodegeneration. NO is also reported to create some sort of a cycle where it triggers A*β* deposition as discussed above, which in turn activates resident immune cells. These glial cells secrete NO and this continuous cycle is thought to have a detrimental impact on AD patients. Other forms of NOS are also upregulated in AD patients, indicating that these enzymes are very instrumental in the pathogenesis of this disease.

## 6. Therapeutic Strategies Targeting NO Signaling Pathways

Alzheimer's disease, one of the leading forms of dementia, continues to elude the scientific fraternity, particularly with respect to pathophysiology given its multifaceted nature of its origin. Although research to work out the molecular mechanisms underlying the cause and the progression of the disease is advancing rapidly, we are yet to do as much with respect to therapeutics. Currently, there is no absolute cure for AD: the few drugs available to clinicians [9] do alleviate the clinical symptoms. Therefore, it is more pertinent than ever that we devise therapeutic strategies that can arrest the disease in total absoluteness. We highlight both the upstream mechanisms that upset the NO homeostasis and the downstream molecular targets, which are the subject of contention in the context of neurodegeneration, which might be useful as therapeutic targets.

Given the fact that NO and its classical metabolites have potentially been implicated in the pathogenesis of AD [19, 60], it is reasonable that drug targeting signaling mechanisms where NO pathologically might lead to neuronal death be thoroughly explored, particularly iNOS upregulation. In addition, capitalizing on the neuroprotective roles of NO as well presents a strategy worth looking into [63].

To date, the most investigated hallmarks integral to AD pathogenesis are A*β* deposition, neurofibrillary tangles, inflammation, and oxidative/nitrosative stress. In each one of them, NO acts to both suppress (neuroprotective) and aggravate (neurotoxic) these factors. Such a biphasic model presents an opportunity to identify and modulate receptor molecules through which NO mediates its biological effects.

Several mechanisms through which NO regulates and mediates neurotoxicity and neuroprotection have been proposed as possible targets for therapeutic schools of thought [64, 78]. Such targets include but are not limited to calcium channel blockers, iNOS inhibitors, molecules that can clear amyloid peptides, NO bioavailability boosters, and antioxidants [78, 79].

### 6.1. NOS Inhibition

**Figure 2:**
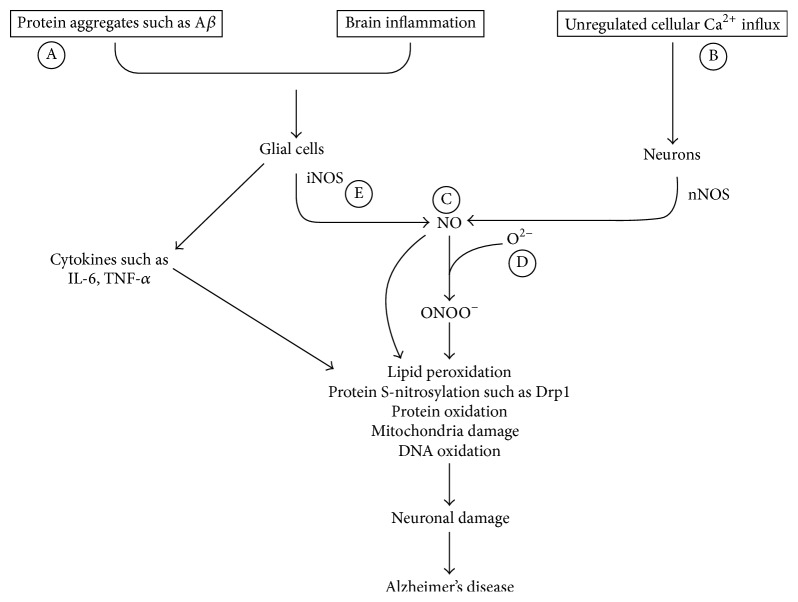
The proposed AD therapeutic approaches targeting NO pathway. (A) Vaccines and agents that could clear *β*-amyloid proteins. (B) Calcium channel blockers. (C) Molecules that can increase bioavailability of NO such as NO mimetics. (D) Antioxidants. (E) iNOS inhibitor.

It has been scientifically proven that all the three isoforms of NOS have a role in the progression of AD. Therefore, targeting them to arrest the advancement of the disease is rather resoundingly relevant. Of particular interest is iNOS, an enzyme responsible for production of copious NO levels manifested in inflammation [80] (Figure 2). However, targeting such an enzyme that exhibits such a critical role in body physiology requires a high level of specificity to circumvent any possible collateral damage that might come along with the nonspecific inhibition such as targeting enzyme sites: arginine, heme, and tetrahydrobiopterin (BH4) sites [78].

Moreover, rather than directly targeting the enzymes, scientists have taken the higher road of trying to subdue the inflammatory insults that result in profuse NO secretion. Compounds that target and inhibit reactivation of glial cells have been investigated [80]. Thippeswamy and his colleagues investigated compound 1400 W, an inhibitor of NOS. This compound was found to downregulate excitation of glial cells as well as suppress potassium (Kir 4.1) receptors partially. It was also found to act on glutamate transporter-1 (GLT-1) levels but insignificantly. It was concluded that the compound was much more effective against gliosis but with a higher level of effectiveness could be realized when supplemented with an anti-inflammatory agent [80]. Although targeting the glial cells presents a safer strategy, manipulating the enzymes would be a much more effective course of action. Therefore, it is imperative to do more work to characterize and single out structural and molecular differences among the three isoforms so as to design highly specific compounds.

### 6.2. A*β* Aggregation and Clearance

It has also been suggested that inhibiting aggregation of A*β* aggregation plaque formation could be a viable option for drug development given the central role played by this protein in the AD pathogenesis [71, 81]. A compound such as resveratrol, which is considered a strong antioxidant, has been shown to possess both anti-inflammatory effects and antiaging effects [81]. It was also found out that it can suppress A*β* aggregation in vitro [81]. Therefore, given its anti-inflammatory and antiaging potential, it is a strong candidate for drug development.

Additionally, vaccines targeting beta amyloid proteins, amyloid-*β*, *α*-synuclein, and tau protein, have been considered [82–85]. These monoclonal antibodies directed to amyloid protein epitopes have been found not only to lower A*β* but also to inhibit formation of tau protein as well and, in turn, suppress activation of glial cells. The prospects might as well cause premature excitement due to such promising findings. However, most of these findings have been obtained using transgenic models and so the results cannot be extrapolated or generalized to human participants. On the other hand, clinical trials for some of the promising vaccine candidates have been conducted, only to be terminated due to associated adverse effects [86, 87]. The other target we propose is the calcium channels since calcium plays a role in NO release by nitrergic nerves through NMDA receptor stimulation by glutamate.

## 7. Conclusion

Free radicals (ROS/RNS) are produced by normal metabolism and are involved in various physiological and pathological conditions [88]. There is a homeostatic balance between free radical production and scavenging by antioxidants. Oxidative stress and nitrosative stress occur when this equilibrium is breached because the excessive ROS/RNS concentrations cannot be neutralized or countervailed by the existing antioxidant systems [88]. Consequently, macromolecules fundamental to the normal functioning of the body such as lipids and proteins can be oxidized or nitrosated depending on the species at play. NO is one of those remarkable gaseous free radicals produced endogenously and has multiple physiological effects ranging from vasodilation to neuromodulation and inflammation. NO essentially mediates its inflammatory effects either directly by reacting with its targets or through reacting with superoxide. The latter causes S-nitrosylation of body biopolymers. NO-mediated oxidative stress has been implicated in a host of rather irreparable cellular aberrances such as cancer, arthritis, and neurodegenerative disorders. Therefore, to manage disorders associated with oxidative stress, therapeutic interventions need to be designed to target the signaling pathways modulated by NO and its downstream conventional metabolites.

The development of drugs aimed at downregulating oxidative stress and improving NO bioavailability in AD patients and other neurodegenerative diseases is a viable option and so is imperative. These approaches include antioxidants and anti-inflammatory mediators, calcium channel inhibitors/blockers and agents that could aid in A*β* clearance, and molecules which prevent and scavenge high concentrations of free radicals.
